# Cyclin A1 promoter hypermethylation in human papillomavirus-associated cervical cancer

**DOI:** 10.1186/1471-2407-6-55

**Published:** 2006-03-08

**Authors:** Nakarin Kitkumthorn, Pattamawadee Yanatatsanajit, Sorapop Kiatpongsan, Chureerat Phokaew, Surang Triratanachat, Prasert Trivijitsilp, Wichai Termrungruanglert, Damrong Tresukosol, Somchai Niruthisard, Apiwat Mutirangura

**Affiliations:** 1 Molecular Biology and Genetics of Cancer Development Research Unit, Faculty of Medicine, Chulalongkorn University, Rama IV., Bangkok 10330, Thailand; 2 Department of Microbiology, Faculty of Medicine, Chulalongkorn University, Rama IV., Bangkok 10330, Thailand; 3 Department of Obstetrics and Gynecology, Faculty of Medicine, Chulalongkorn University, Rama IV., Bangkok 10330, Thailand; 4 Department of Anatomy, Faculty of Medicine, Chulalongkorn University, Rama IV., Bangkok 10330, Thailand

## Abstract

**Background:**

The aim of this study was to evaluate epigenetic status of *cyclin A1 *in human papillomavirus-associated cervical cancer. Y. Tokumaru *et al*., *Cancer Res ***64**, 5982-7 (Sep 1, 2004)demonstrated in head and neck squamous-cell cancer an inverse correlation between *cyclin A1 *promoter hypermethylation and *TP53 *mutation. Human papillomavirus-associated cervical cancer, however, is deprived of TP53 function by a different mechanism. Therefore, it was of interest to investigate the epigenetic alterations during multistep cervical cancer development.

**Methods:**

In this study, we performed duplex methylation-specific PCR and reverse transcriptase PCR on several cervical cancer cell lines and microdissected cervical cancers. Furthermore, the incidence of *cyclin A1 *methylation was studied in 43 samples of white blood cells, 25 normal cervices, and 24, 5 and 30 human papillomavirus-associated premalignant, microinvasive and invasive cervical lesions, respectively.

**Results:**

We demonstrated *cyclin A1 *methylation to be commonly found in cervical cancer, both in vitro and in vivo, with its physiological role being to decrease gene expression. More important, this study demonstrated that not only is *cyclin A1 *promoter hypermethylation strikingly common in cervical cancer, but is also specific to the invasive phenotype in comparison with other histopathological stages during multistep carcinogenesis. None of the normal cells and low-grade squamous intraepithelial lesions exhibited methylation. In contrast, 36.6%, 60% and 93.3% of high-grade squamous intraepithelial lesions, microinvasive and invasive cancers, respectively, showed methylation.

**Conclusion:**

This methylation study indicated that *cyclin A1 *is a potential tumor marker for early diagnosis of invasive cervical cancer.

## Background

Cervical cancer (CC) is an important health problem and is a leading cause of cancer mortality worldwide in women. [1] When exposed to and infected by one of the high-risk human papillomaviruses (HPV), vulnerable cervical epithelium may enter a complex multistep process and develop an invasive carcinoma. [2-4] The spectrum of histologic alterations during the intricate processes of multistep carcinogenesis can be classified as premalignant lesions, including low-grade and high-grade squamous intraepithelial lesions (SILs), and malignant invasive cervical cancers. [5] Despite its strong association with CC, HPV infection alone is not sufficient for the cervical epithelium to fully develop an invasive cervical cancer. Persistent HPV infection contributes to the development of SILs, with viral oncoproteins facilitating the dysregulation of cellular proliferation and the apoptotic process. However, additional accumulation of mutations, as well as epigenetic alterations in the crucial oncogenes and tumor suppressor genes, is required before these premalignant lesions fully transform into invasive cancers. [6]

The aim of this study was to evaluate DNA methylation status of *cyclin A1 *(*CCNA1*) in HPV-associated CC. *CCNA1*, a second A-type cyclin, has been shown to be essential for entry into metaphase of male meiosis I[7,8] Consistent with this function, *CCNA1 *is highly expressed in testis and hematopoietic progenitor cells, but is present at low levels in most other tissues. [9] No phenotype other than male infertility has been reported in mice lacking *CCNA1*. [10] Surprisingly, several lines of evidence suggest that *CCNA1 *may be a potential epithelial tumor suppressor gene. First, the expression of *CCNA1 *has been demonstrated to be downregulated in several cancers, such as nasopharyngeal carcinoma and head and neck squamous-cell cancer (HNSCC). [11-13] Second, CCNA1 plays an important role in DNA double-strand break repair following radiation damage by activation of the non-homologous end-joining process that confers DNA stability. [14] Finally, the promoter, similar to several key tumor suppressor genes, is frequently hypermethylated in colon cancer and HNSCC. [13,15]

Expression of *CCNA1 *has been shown to be correlated with the activation of *TP53*. In a HNSCC model, there is an inverse relationship between *CCNA1 *promoter methylation and *TP53 *mutation status in HNSCC tissues. [13] Similar to HNSCC, the majority of CC is of squamous cell origin and its molecular carcinogenesis strongly correlates with impaired TP53 function. [16-18] However, unlike HNSCC, the functional loss of TP53 in CC is not ascribed to gene mutation, but is processed by viral and host protein-protein interaction. CC is strongly associated with infection by high-risk HPV types and its oncoprotein E6 has the ability to associate with and neutralize the function of TP53. [17,18] E6 binds to TP53 and catalyzes multi-ubiquitination and degradation of TP53. Consequently, the majority of CC cells have a wild-type *TP53*, but the protein levels are decreased. Therefore, in comparison with HNSCC, it was of interest to determine if *CCNA1 *is methylated in HPV-associated squamous cell CC.

## Methods

### Cell lines and tissue samples

SiHa and two HeLa CC cell lines from different sources were grown in Dulbecco's modified Eagle's medium supplemented with 10% fetal bovine serum. All three cells were purchased from ATCC. SiHa, HeLa (S), and HeLa (K) were grown and maintained in laboratories of Dr. Ponglikitmongkol M, Mahidol University, Dr. Gutkind JS, NIH, USA and Dr. Ruxrungthum K, Chulalongkorn University, respectively.

With approval of ethical committee, faculty of medicine, Chulalongkorn university, normal cervical tissues, cancer tissues and blood samples were obtained and prepared as previously described. [19,20] Cervical tissues were obtained by punch biopsy of lesions under direct visualization or under colposcopic examination. Specimens were divided in two. The first sample was submitted to routine histological examination, and the second was reserved for DNA isolation. Blood samples were obtained by venipuncture from CC patients and healthy blood donors. All HPV-positive premalignant lesions were exfoliated cells, selected from routine cytological screening. In brief, cervical cells were collected with a cervical sampler (Digene Corporation, Gaithersburg, MD, USA) using the cervical cytobrush technique, and were divided into three parts. The first was reserved for routine cytological diagnosis. The second was tested for the presence of high-risk HPV (types 16, 18, 31, 33, 35, 39, 45, 51, 52, 56, 58, 59, and 68) DNA by Hybrid Capture 2 (Digene Corporation, Gaithersburg, MD, USA). [21] In cases of positive high-risk HPV and complete histological tissue evaluation, the third part was subjected to *CCNA1 *methylation analysis. DNA extraction was performed using Tris/SDS and proteinase K at 50°C overnight, followed by phenol/chloroform extraction and ethanol precipitation.

Cervical biopsy specimens and Papanicolaou smears were examined and reviewed by at least two gynecologic pathologists to ensure good quality control of the final pathology results. All CCs contained 20–95% malignant cells. The histological diagnoses distinguished among normal epithelium, low-grade SILs, high-grade SILs, microinvasive and invasive cancer. In case of invasive cancer, only those samples classified as squamous-cell lesions were used for further analysis.

Additional six OTC-embedded frozen CCs and five normal cervices, obtained from hysterectomy specimens, were microdissected as previously described.^22 ^Histologically normal epithelium, connective tissue and malignant cells were subjected to *CCNA1 *methylation and expression studies.

### HPV detection and typing

HPV *L1*, *E6 *gene amplification and dot blot hybridization were performed as previously described[19,22,23] Briefly, each L1 amplification reaction contained the L1 degenerate primers MY11 and MY09. The E6 reactions contained WD72, WD66, WD154, WD67 and WD76. Both reactions were used to amplify genomic DNA during 40 PCR cycles. To analyze the amplicons for the presence of high-risk HPV, we applied dot blot hybridization using the HPV type-specific oligo probes, WD170, WD132, RR1, RR2, WD103, WD165, WD, consensus L1, MY12/13, WD126, WD128, MY16, WD133/134, MY14 and WD174. The membranes were subjected to analysis by a phosphoimager. Results for L1 and E6 dot blots were scored independently. Duplicate filters were prepared for all specimens.

### Sodium bisulfite modification and duplex methylation-specific PCR (MSP)

The DNA samples were subjected to bisulfite treatment. [24,25] Briefly, 2 μg of genomic DNA was denatured with NaOH (final concentration 0.2 M). Subsequently, 10 mM hydroquinone and 3 M sodium bisulfite were added and incubated at 50°C for 16 h. The modified DNA was then purified using Wizard DNA purification resin (Promega, Madison, WI, USA) followed by ethanol precipitation. Duplex MSPs were performed to identify the *CCNA1 *methylation status of all samples. The duplex PCR mixtures contained 10× PCR buffer (Qiagen, Chuo-ku, Tokyo), deoxynucleotide triphosphates (0.2 mM), primers CCNA1metF, CCNA1metR, CCNA1unmetF and CCNA1unmetR (final concentration 0.4 μM each per reaction) (Table 1), 1 U of HotStarTaq (Qiagen, Chuo-ku, Tokyo) and bisulfited DNA (80 ng). The amplification reaction was carried out for 30 cycles in a 2400 Perkin Elmer thermal cycler. Then 10-μl aliquots of the PCR products were stained with cyber green, run on an 8% non-denaturing polyacrylamide gel. The band intensity was visualized and measured by using a phosphoimager.

### RNA preparation and analysis

Expression of *CCNA1 *in the CC cell lines was examined by RT-PCR. Total RNA was extracted using the TRIZOL reagent (Invitrogen, Singapore) according to the manufacturer's specifications and 5 μg of each sample was subjected to cDNA synthesis using MMLV reverse transcriptase (Fermentas, Hanover, MD, USA). PCR mixtures contained 10× PCR buffer, 0.2 mM dNTPs, 0.4 μM each of primers CCNA1cDNAF and CNA1cDNAR, 1 U of HotStartaq and 80 ng cDNA. *GAPDH *served as the internal control (Table 1). Aliquots of 10 μl of the PCR products were subjected to electrophoresis on a 2% agarose gel stained with ethidium bromide on preparation, and were visualized by a UV trans-illuminator.

### Bisulfite genome sequence analysis

Some *CCNA1 *methylation-positive CCs were selected for sequence analysis. The bisulfited DNAs were amplified using CCNA1cloningF and CCNA1cloningR (Table 1). The amplified fragments were cloned using the PGemT easy vector and sequenced.

## Results

The aim of this study was to determine if the *CCNA1 *promoter is methylated in CC and to elucidate how the epigenetic alteration occurs during multistep CC development. The experiments conducted comprised of: first, establishment of *CCNA1 *MSP; second, identification of the methylation status and correlation with expression in CC cell lines, normal cervix and CC; and finally, investigation of the frequency of methylation in normal tissues, high-risk HPV-associated low SILs, high SILs, microinvasive and invasive squamous cell CC.

### CCNA1 methylation in CC cell lines

**Figure 1:**
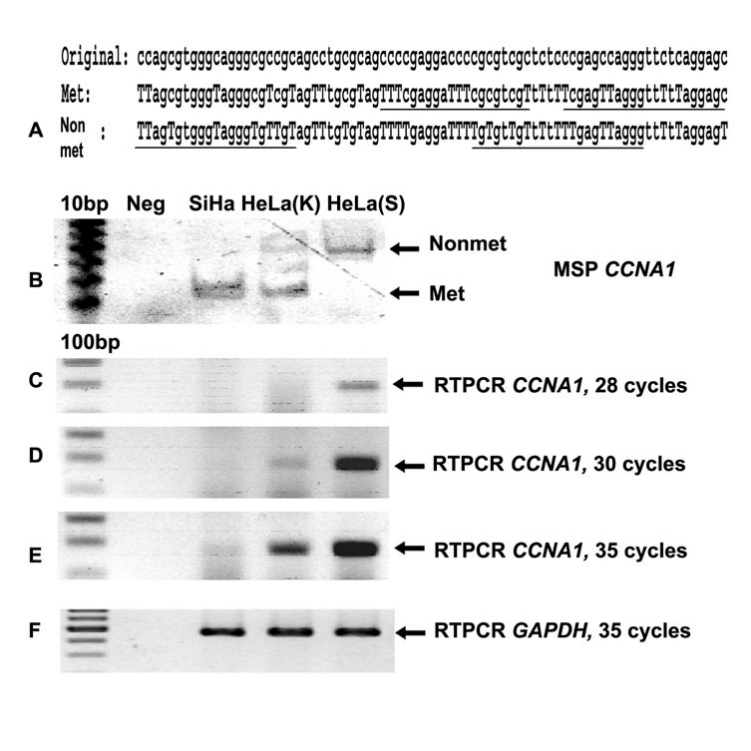
Schematic representation of inverse correlation between promoter methylation and expression of *CCNA1 *in CC cell lines. (A) Diagram of methylated and non-methylated sequences after bisulfite modification covering the area of both primers (underlined) in the promoter region of *CCNA1*. M, DNA size marker. Top panel, 10-bp ladder; bottom four panels, 100-bp ladder. Neg, negative. (B) Duplex MSP analysis of cell lines. Upper and lower arrows indicate non-methylated and methylated amplicons, respectively. MSP, methylation-specific PCR. (C-E) RT-PCR of the *CCNA1 *gene after 28, 30 and 35 cycles, respectively. (F) RT-PCR of the *GAPDH *gene as an internal control.

Duplex MSP for *CCNA1 *was designed according to the sequence in Figure 1A. The methylated sequence comprised of 46 bp and the non-methylated sequence, 67 bp, shown as the lower and the upper amplicons, respectively.

Previously, Carsten Müller-Tidow et al. [26,27] extensively studied the role of *CCNA1 *methylation and found that *CCNA1 *was methylated in several non-expressing tumor cell lines, including HeLa. To confirm this particular finding in CC cell lines, we investigated methylation and expression in HeLa and SiHa cells. Our preliminary study in HeLa, HeLa(S), revealed complete non-methylation, which contradicts the previous report (Fig. 1B). To settle this controversy, we attempted to further evaluate additional CC cell lines, including HeLa(K) grown in a different laboratory, and SiHa. The result confirmed the Carsten Müller-Tidow et al. [26,27] finding, in that the majority of Hela(K) cells, as well as all SiHa cells, were hypermethylated. CCNA1 RT-PCR confirmed the inverse relation between DNA methylation and gene expression. CCNA1 RNA levels were high, intermediate and low in HeLa(S), HeLa(K) and SiHa cells, respectively (Fig. 1). These data indicate that *CCNA1 *methylation is common in CC cell lines and its physiological role is to decrease gene expression. The absence of methylation in HeLa(S) might indicate a demethylation process that occurs under different cell culture and maintenance conditions.

**Figure 2:**
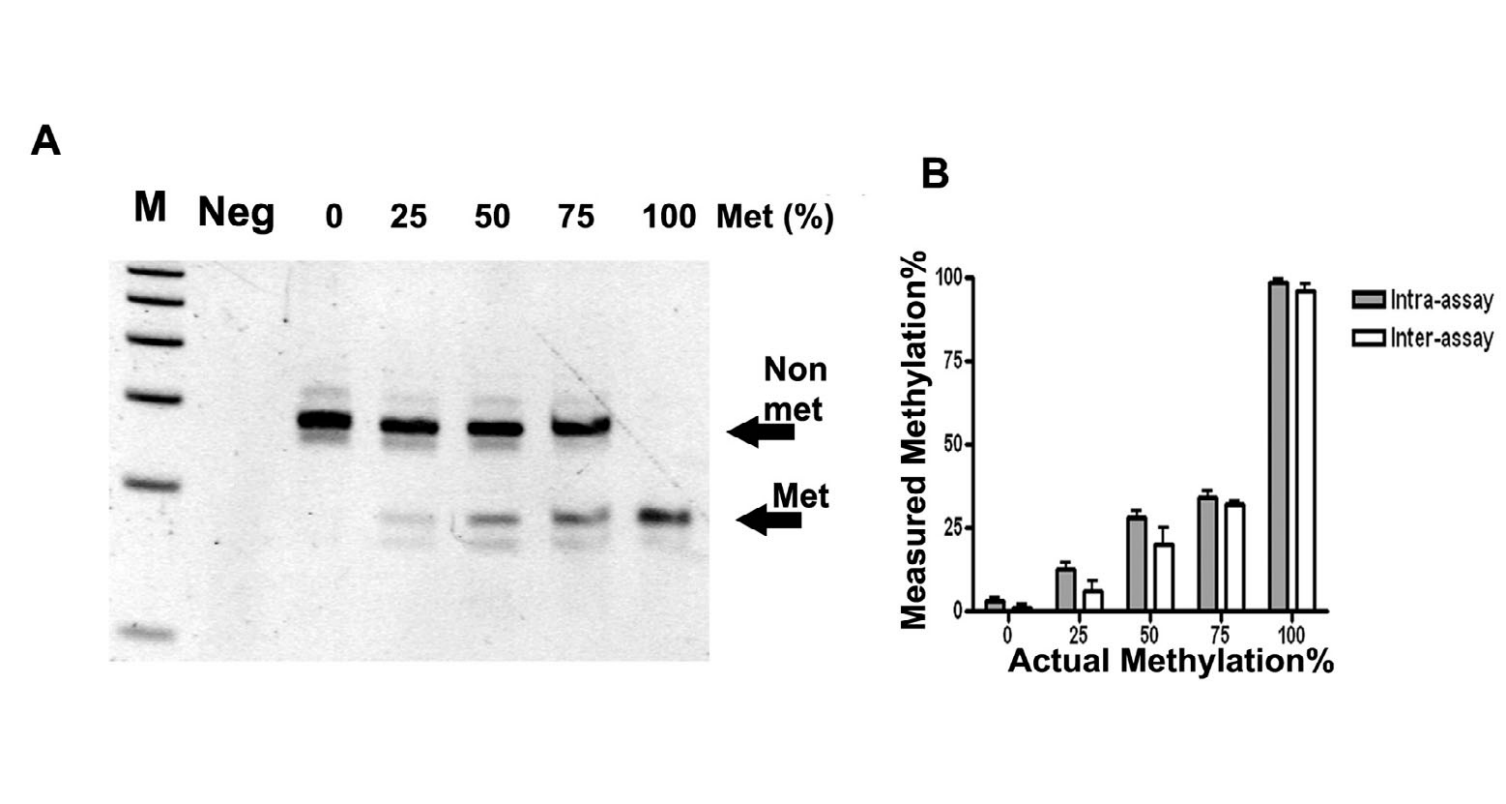
Intra- and inter-assay variation of the duplex MSP. (A) Duplex MSP of a mixture of *CCNA1 *complete and non-methylated CC cell lines, SiHa and HeLa(S), respectively. M, DNA size marker; Neg, negative; 0, 25, 50, 75, 100 Met (%) represent the proportion of SiHa DNA in the mixture, varied from 0 to 100%, respectively. The upper and lower bands are non-methylated and methylated bands, respectively, indicated by labeled arrows. (B) Graphical comparison between measured *CCNA1 *methylation, percentage intensity of methylation amplicon (x-axis), and actual methylation, the proportion of SiHa DNA (y-axis). The bar height indicates the mean and error bars, T, represent standard deviation (SD) across experiments.

We validated the reliability of this duplex MSP by performing calibration experiments using SiHa mixed with HeLa(S), CCNA1 completely hypermethylated and non-methylated cells, respectively (Fig. 2A). With at least three replicates for each experiment, the result demonstrates the consistency of the current approach, with minimal intra- and inter-assay variations (Fig. 2B). It is noteworthy that the correlation between measured and actual CCNA1 methylation percentages was not linear, but exponential.

### CCNA1 methylation and expression in cervical tissues

**Figure 3:**
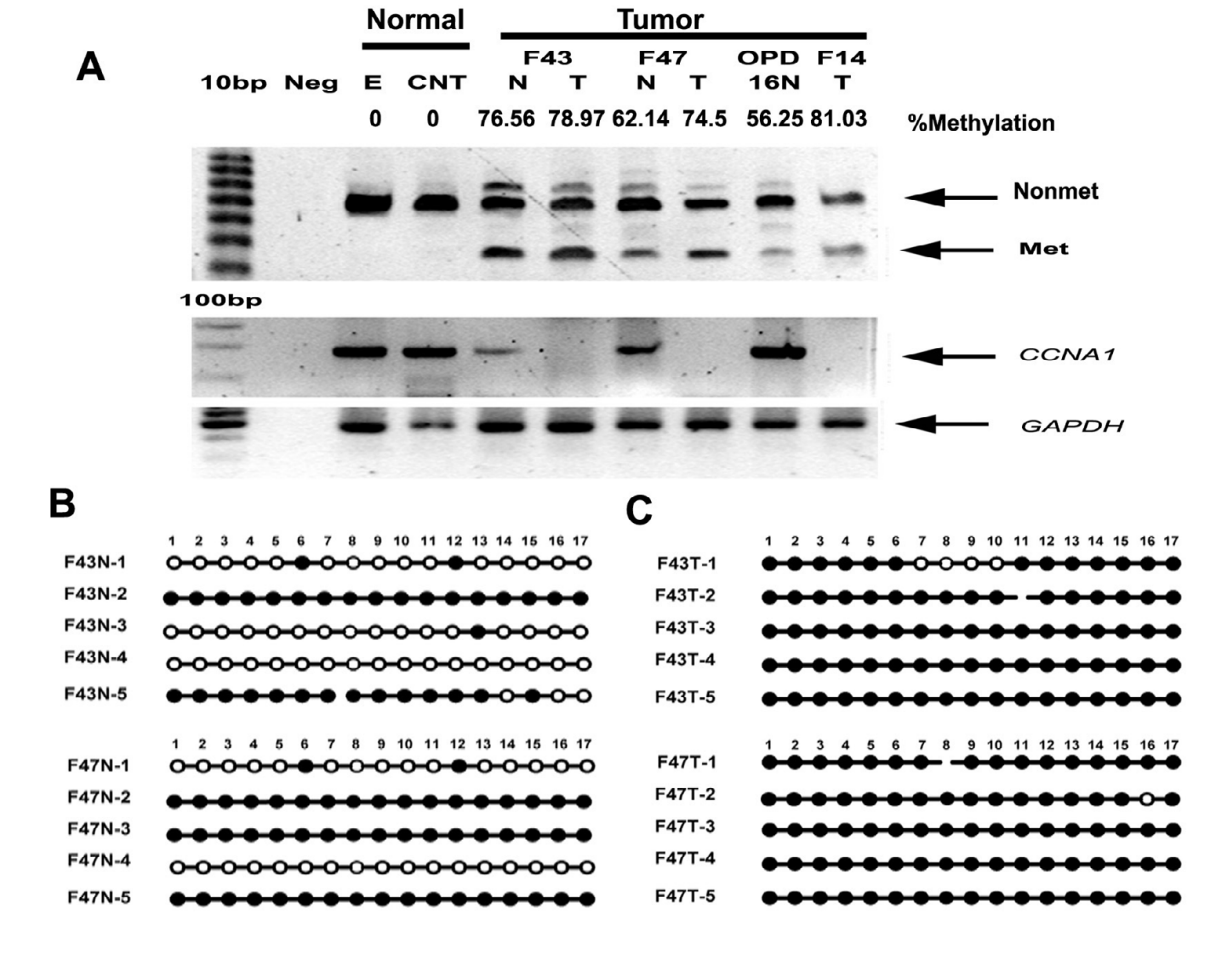
*CCNA1 *methylation and expression in microdissected cervical tissues. (A) Duplex MSP and *CCNA1 *PCR; E and CNT are epithelium and connective tissue cells from normal cervix; N and T are adjacent histological normal and cancer cervical epithelium from CC, respectively. Arrows indicate non-methylated, methylated, *CCNA1 *cDNA and *GAPDH *cDNA, respectively. (B) Bisulfite sequencing at the *CCNA1 *promoter, with circles denoting the methylation status of each selected clone. Black and white circles are methylated CG dinucleotides, and non-methylated CpG dinucleotides and TG dinucleotides, respectively.

The discovery of an inverse correlation between CCNA1 methylation and expression in CC lines suggested possibility of the same situation in vivo. To test this hypothesis, we evaluated the epigenetic control in vivo. Six frozen OTC-embedded CCs and five normal cervices were microdissected and subjected to duplex MSP and CCNA1 RT-PCR. Figure 3 shows examples of typical in vivo results. First, whereas no methylation could be observed, CCNA1 mRNA was discoverable by RT-PCR in normal cervix from both epithelium and connective tissue cells (Fig. 3A). In contrast, epigenetic control was detectable in cervical epithelia of CC patients from both malignant cells and adjacent histologically normal cervical epithelia. Nonetheless, in matched cases, a higher degree of methylation could be demonstrated in cancer than in normal cells. From all CCs, no CCNA1 mRNA was detectable. Interestingly, even if methylation was detected, CCNA1 was expressed in malignancy-adjacent histologically normal cervical tissues. Moreover, an inverse correlation between the methylation level and mRNA quantity was observed. CCNA1 expression in methylated malignancy-adjacent histologically normal cervical epithelium may be due to normal cell contamination or partial methylation at the promoter according to CC multistep progression. Whereas complete methylation could be observed in most cancer cells, partial and non-methylated CCNA1 was discovered in the adjacent epithelia (Fig. 3B). In conclusion, this experiment evaluating cervical tissue in vivo led to three conclusions. First, *CCNA1 *methylation was exclusively associated with cervical carcinogenesis. Second, the epigenetic alteration occurred earlier than morphological transformation of the cellular phenotype. Finally, methylation may play a role in this gene inactivation.

### CCNA1 methylation incidence during multistep cervical carcinogenesis

**Figure 4:**
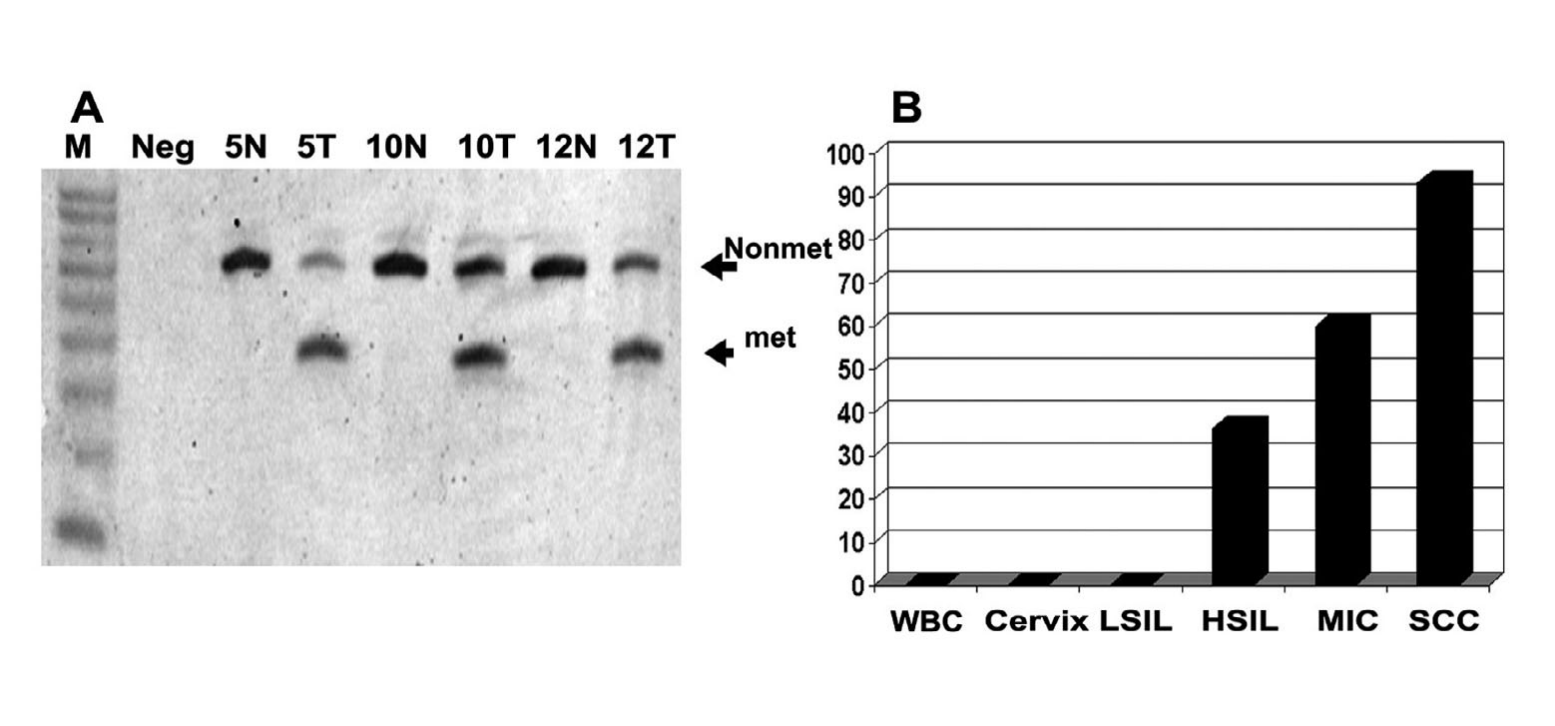
Schematic representation of methylation-specific PCR in CC. (A) PCR analysis of CC: M, DNA size marker; Neg, water; N and T, matched normal cervices and tumors, respectively. (B) Bar graph demonstrating the frequency of DNA methylation. Numbers on the y-axis are the percentage of positive methylation cases. Sample types are on the x-axis. WBC, normal cervix, Low-grade SIL, High-grade SIL, microinvasive cancer and squamous cell CCs number are 43, 25, 13, 11, 5, and 30, respectively. The methylation frequencies of each tissue type are represented by the height of each rectangular bar.

Cervical intraepithelial neoplasia provides a crucial model to study the multistep process of carcinogenesis. Therefore, we evaluated the frequency of CCNA1 methylation in several cervical epithelial tissues with a distinctive degree of malignant transformation, normal cervix, CIN, microinvasive and CC, respectively. We selected 43, 25 and 30 cases of white blood cells (WBC), normal cervical biopsies and invasive CCs, respectively (Table 2). Among these samples, 13 WBC samples and 6 normal cervical samples, located at least 3 cm from the tumor margin and showing the absence of HPV DNA, originated from CC patients. For all cases, when a methylated amplicon was visible and the methylation percentage measured exceeded 5%, the test was deemed positive. All selected CCs were squamous and positive for HPV. Of the cases, 24 harbored HPV type 16, 4 had HPV type 18 and 2 cases displayed unclassifiable HPV types. Interestingly, a high frequency of methylation was exclusively present in CCs, i.e., 28 cases or 93.3% (Fig. 4A,B and Table 2). To reveal multistep carcinogenesis, we included 24 cases of SILs and 5 microinvasive cancers from exfoliated cervical cells. All cases were positive for oncogenic HPV, analyzed by Hybrid Capture 2. Whereas 60% and 36.6% of the microinvasive cancers and high SILs, respectively, demonstrated *CCNA1 *methylation, none of the HPV-associated low SILs exhibited these epigenetic changes (Fig. 4B and Table 2).

## Discussion

This study demonstrated that: (i) *CCNA1 *promoter hypermethylation in HPV-associated squamous cell CC is unusually common; (ii) it is specific to CC; and (iii) the methylation is more common in invasive phenotypes compared to other histopathological stages during multistep carcinogenesis. This finding identifies both the interesting biology of CC and a potential clinical application of *CCNA1 *methylation as an additional molecular marker for the early diagnosis of invasive CC.

Annual cytology screening has dramatically increased the effectiveness of early CC detection. Nonetheless, additional tests will help to improve the sensitivity and specificity of a single Papanicolaou smear for histological analysis. Recently, testing for oncogenic HPVs has been introduced to aid in the triage of women with atypical squamous cells of undetermined significance (ASCUS). [28] However, because the majority of patients with HPV-associated lesions do not progress to invasive cancer, several studies have attempted to add a panel of tumor suppressor gene methylations to improve the effectiveness of molecular cytological diagnosis. [29,30] Since the frequency of *CCNA1 *methylation is high and specific to invasive CC, this gene should be a good candidate to increase the coverage rate for early cancer detection.

In HNSCC, *CCNA1 *promoter hypermethylation is inversely related to *TP53 *mutation. [13] Nonetheless, the frequency of *CCNA1 *promoter hypermethylation in CC is high, whereas the function of TP53 in CC is usually impaired as a consequence of protein degradation induced by binding of the viral E6 protein. [18] This observation may be due to either differences in tissue types or pathophysiological outcomes of *TP53 *between mutations and diminution of the protein function subsequent to E6 binding. We prefer the latter hypothesis, since *TP53 *and *CCNA1 *have been shown to augment each other's expression. [13,14] Consequently, the CCNA1 protein could help to increase physiologic TP53 to counter the function of E6, except for cases of *TP53 *mutation. In other words, alterations of both *CCNA1 *and *TP53 *in HNSCC will be redundant. In contrast, in CC, a decrease in CCNA1 protein should prevent the increment of TP53 that would have compensated for the protein destruction by E6.

Multistep process analysis revealed that *CCNA1 *methylation is remarkably specific for cervical carcinogenesis. The biological function of CCNA1 is to activate DNA breakage repair by mechanisms depending on CDK2 activity and Ku proteins. [14] It is interesting to hypothesize why the genomic instability, triggered by impairment of the CCNA1 function, is crucial as an early event in CC development. Perhaps the rate of spontaneous mutations in cervical epithelial cells is too low to accumulate sufficient malignancy-transformation-dependent oncogene and tumor suppressor gene mutations if the cells possess fully functional CCNA1. Therefore, the frequency of invasive CC devoid of *CCNA1 *methylation is limited.

## Conclusion

This study demonstrates the strong association between *CCNA1 *promoter hypermethylation and invasive HPV-associated CC indicates that this gene could serve as an effective molecular marker. Moreover, our finding, in comparison with previous reports, [13,14] also suggests that there is a possible molecular link between oncogenic HPVs, TP53 and *CCNA1 *promoter hypermethylation.

## Abbreviations

CC: cervical cancer, CCNA1: cyclin A1, SILs: squamous intraepithelial lesions, HPV: *Human papillomavirus*, WBC: white blood cell

## Competing interests

The author(s) declare that they have no competing interests.

## Authors' contributions

NK: Perform all experiments, data analysis and write the article. PY: set up duplex MSP experiment, CP: collecting and HPV analysis of CIN, SK, ST, PT, WT, DT and SN: collect clinical samples and data, AM: Hypothesize, design and analyze the experiments and write the article

## Pre-publication history

The pre-publication history for this paper can be accessed here:



## Figures and Tables

**Table 1:** Oligonucleotide sequences and conditions for PCR analyses

Primer	Sequence	Amplicon size (bp)	Annealing temperature (°C)
CCNA1metF	TTTCGAGGATTTCGCGTCGT	46	53
CCNA1metR	CTCCTAAAAACCCTAACTCGA		
CCNA1unmetF	TTAGTGTGGGTAGGGTGTT	67	53
CCNA1unmetR	CCCTAACTCAAAAAAACAACACA		
CCNA1cloningF	TGGGTAGGGCGTCGTAGTT	196	55
CCNA1cloningR	GCCCCCGACCTAAAAAAA		
CCNA1cDNAF	ATTCATTAAGTGAAATTGTGC	170	47
CCNA1cDNAR	CTTCCATTCAGAAACTTATTG		
GAPDHF	GTGGGCAAGGTATCCCTG	460	52
GAPDHR	GATTCAGTGTGGTGGGGGAC		

**Table 2:** *CCNA1 *methylation and clinico-pathological correlation

Histological characteristics	Total number of cases	*CCNA1 *promoter hypermethylation	
		
		Absent	Present
WBC	43	43	0
Normal cervix	25	25	0
Low-grade SIL	13	13	0
High-grade SIL	11	7	4
Microinvasive cancer	5	2	3
Squamous cell CC	30	2	28
FIGO stage I-IIA	6	0	6
FIGO stage IIB-IV	24	2	22
Grade 1, keratinized type	9	0	9
Grade 2, non-keratinized type	21	2	19

FIGO, International Federation of Gynecology and Obstetrics.
